# A highly predictive autoantibody-based biomarker panel for prognosis in early-stage NSCLC with potential therapeutic implications

**DOI:** 10.1038/s41416-021-01572-x

**Published:** 2021-11-02

**Authors:** Akshay J. Patel, Ti-Myen Tan, Alex G. Richter, Babu Naidu, Jonathan M. Blackburn, Gary W. Middleton

**Affiliations:** 1 Institute of Immunology and Immunotherapy (III), College of Medical and Dental Sciences, University of Birmingham, Birmingham, England UK; 2 Sengenics Corporation, Level M, Plaza Zurich, Damansara Heights, Kuala Lumpur, 50490 Malaysia; 3 Institute of Inflammation and Ageing (IIA), College of Medical Sciences, University of Birmingham, Birmingham, England UK; 4 Institute of Infectious Disease and Molecular Medicine; Department of Integrative Biomedical Sciences; Faculty of Health Sciences, University of Cape Town, Cape Town, South Africa

**Keywords:** Non-small-cell lung cancer, Tumour biomarkers

## Abstract

**Background:**

Lung cancer is the leading cause of cancer-related death worldwide. Surgical resection remains the definitive curative treatment for early-stage disease offering an overall 5-year survival rate of 62%. Despite careful case selection, a significant proportion of early-stage cancers relapse aggressively within the first year post-operatively. Identification of these patients is key to accurate prognostication and understanding the biology that drives early relapse might open up potential novel adjuvant therapies.

**Methods:**

We performed an unsupervised interrogation of >1600 serum-based autoantibody biomarkers using an iterative machine-learning algorithm.

**Results:**

We identified a 13 biomarker signature that was highly predictive for survivorship in post-operative early-stage lung cancer; this outperforms currently used autoantibody biomarkers in solid cancers. Our results demonstrate significantly poor survivorship in high expressers of this biomarker signature with an overall 5-year survival rate of 7.6%.

**Conclusions:**

We anticipate that the data will lead to the development of an off-the-shelf prognostic panel and further that the oncogenic relevance of the proteins recognised in the panel may be a starting point for a new adjuvant therapy.

## Introduction

Worldwide, lung cancer is the leading cause of malignancy-related death in men and the second in women. Only 18% of patients at initial presentation are suitable for curative treatment, mainly surgical resection. The overall 5-year survival is 55%, 35% and 15% for stage 1, 2 and 3 cancers, respectively [1, 2]. However, there are still a considerable proportion of patients with resectable lung cancers who relapse very quickly post resection. The behaviour of these cancers does not obey the expected outcomes based on prognostic scores such as the tumour node metastasis (TNM) staging system. The mainstay of treatment for early-stage non-small cell lung cancer (NSCLC) is radical surgery. Stereotactic radiotherapy can be employed for local disease control in patients who are unfit for surgery, but these cases are at higher risk for recurrence [3]. Adjuvant platinum-based chemotherapy has demonstrated an absolute survival benefit of 5% compared to surveillance alone; however, little progress has been made in this area in the past 10 years. The future of adjuvant therapy will involve multi-modality treatment with targeted molecular agents and immunotherapy [3]. Multi-modality therapy is not without associated morbidity; thus, selecting patients who are biologically most at risk of post-operative recurrence is a major clinical need.

Autoantibody (AAb) profiling is a promising approach that incorporates the immune recognition of a myriad of aberrant cancer proteins into a single diagnostic test. AAbs reflect the initial humoral immune response against a tumour and their increased levels can be detectable months to years prior to clinical evidence of a primary tumour [4] or indeed recurrence post resection of a primary tumour. While the mechanisms involved in the production of AAbs in cancer patients remain speculative, AAbs are well known to be sensitive biomarkers in the detection and surveillance of many types of tumours. Gnjatic and colleagues developed protein microarrays to assay the serological response of cancer patients to tumours (serological expression cloning, SEREX) [4]. These high-density protein microarrays, in which proteins are immobilised in their natural conformations, allow the functional testing of thousands of proteins simultaneously, thus increasing the chance of the discovery of new AAb signatures [5]. Building on this work and principle, we utilised the Sengenics Immunome™ Protein Array [Sengenics, Singapore] containing 1627 proteins, to screen sera from a total of 157 non-small cell lung cancer (NSCLC) patients across two independent cohorts. We set out to identify a high-risk sub-group of surgically resectable NSCLC patients who may benefit from adjuvant therapy, and explore the biological significance of the identified biomarkers along with information relevant to therapeutic application. We implemented a bespoke machine-learning approach in order to investigate the utility of using the pre-resection samples in the context of malignancy to identify sera-based proteomic changes specifically associated with outcome in NSCLC following surgery.

## Materials and methods

Collaborating clinicians and principal researchers prospectively recruited patients involved in our study across two major tertiary sub-specialty centres in the midlands regions of England, UK as part of a large historical observational study (CLUB) between 2010 and 2015. All patients underwent curative NSCLC (adenocarcinoma or squamous cell carcinoma only) resection (stage I–IIIa disease) at two major thoracic surgical units in England. Patients not meeting this inclusion criterion or who had any other previous malignancy were excluded from our study. Inadequate serum sample (<1 ml), non-cancer-related deaths, use of neoadjuvant chemotherapy or positive pathological resection margins were excluded from this study. All participants provided informed consent to participate in future translational studies when they were initially recruited, previously approved by the West Midlands—Solihull Research Ethics Committee (Cancer of the Lung Biomarkers (CLUB): REC reference: 04/Q2704/34). The study had National Cancer Research Network (NCRN) approval and was an NCRN portfolio study. Patients were diagnosed by routine pathological examination of their excised primary tumour and staged according to the TNM staging system for NSCLC according to the International Association for the Study of Lung Cancer (IASLC) guidelines (8th Edition) [6].

### Study design

A total of 157 study participants’ (NSCLC stage I–IIIa) pre-operative serum samples were utilised in the proteomics analysis, taken from a large repository of trial patients. A sample size calculation, undertaken to achieve a power of 95% was determined based on the standard deviations of each protein in the immunome array. A random set of patients was selected from the total study participants (investigators were blinded to clinical metadata) in order to train the machine-learning model and subsequently tune the model hyperparameters using *k*-fold cross-validation. This training cohort is known as cohort 1. A smaller independent, second cohort was randomly selected to provide an unbiased evaluation of the final model and validate the model (cohort 2). Cohort sizes were determined using a stratified random sample-based approach to split the overall dataset. For reasonably sized datasets (*n* > 100), this commonly used approach in machine-learning settings has been shown to be close to optimal when allocating 66–70% of the samples to the training set (cohort 1) [7].

### Study cohorts

**Fig. 1:**
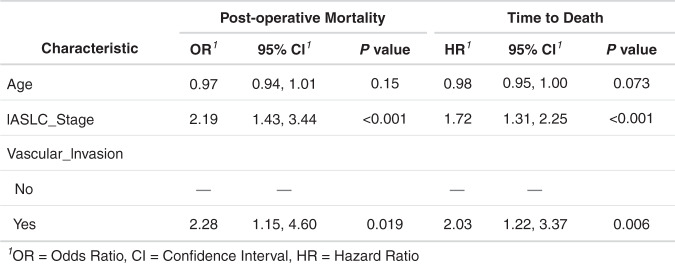
Multivariate analysis using logistic and Cox proportional hazards regression to assess outcome in terms of post-operative mortality and time to death, respectively. Stepwise backward elimination was employed to remove the least significant independent predictors. All variables (age, gender, histology, IASLC stage, nodal status, lymphovascular invasion and adjuvant chemotherapy use) were entered into the model and successively removed depending on significance in the model.

Cohort 1 consisted of 111 NSCLC patients (65 survivors, 46 non-survivors). Cohort 2 consisted of 46 NSCLC patients (27 survivors, 19 non-survivors). Survivors were defined as patients who were alive and recurrence-free at follow-up. The median follow-up of the entire recurrence-free population was 1825 days (range 1195–2555 days). Non-survivors were defined as patients who died from post-operative recurrence within a median of 365 days. The participant characteristics are summarised in Supplementary Table (S1). There was no significant difference between cohorts 1 and 2 in terms of age, gender, histology, stage, vascular invasion, need for adjuvant therapy and overall survival (assessed using Wilcoxon’s rank-sum test and Fisher’s exact test for non-parametric continuous data and categorical data, respectively). There was a higher preponderance of adenocarcinomas in cohort 2 (60.9 versus 49.5%), and a higher preponderance of squamous cell carcinomas in cohort 1 (50.5 versus 39.1%). The survival distribution of the total study population is displayed in Fig. 1. Cox proportional multivariate hazards analysis identified the IASLC stage and the presence of lymphovascular invasion as significant independent negative prognostic risk factors (hazard ratio (HR) 1.72, *p* < 0.001 and HR 2.03, *p* = 0.006, respectively); histology was not significant.

All samples were assayed using the Sengenics Immunome Protein Array containing 1600+ proteins spotted in quadruplicates (Sengenics, Singapore).

### Sample collection

Serum samples were taken at enrolment or prior to surgery and immediately pseudonymised so as to blind investigators to endpoints. Samples were collected from all participants in a starved state to maintain uniformity. A sample of 7 ml whole venous blood was taken into standard collection tubes and allowed to clot for 2 h. Samples were centrifuged at 3000 × *g* for 20 min. Serum was then carefully aspirated, divided into aliquots and stored at −80 °C [8].

### Protein Immunome AAb assay

Serum samples were thawed, mixed by vortexing and any precipitate was pelleted by centrifugation (13,000 × g, for 3 min). Aliquots of each sample (11.25 µl) were then diluted 400-fold into Serum Assay Buffer (SAB; 0.1% v/v Triton, 0.1% w/v bovine serum albumin (BSA) in phosphate-buffered saline; 20 °C), giving a final volume of 4.5 ml.

Replica Immunome protein array slides were removed from storage buffer and washed in 200 ml cold SAB on an orbital shaker (50 RPM, 5 min). Each slide was then placed array side up in a hybridisation chamber and incubated with individual diluted sera (4.5 mL) on a horizontal shaker for 2 h at 20 °C, with gentle agitation. Each protein array slide was then rinsed briefly twice with 30 mL SAB, followed by immersion in 200 mL of SAB buffer for 20 min at room temperature with gentle agitation. Each slide was then incubated with a detection antibody (20 μg/ml Cy3-labelled anti-human IgG in SAB) for 2 h at room temperature with gentle agitation, rinsed briefly with SAB buffer and then washed three times in SAB for 5 min at room temperature. Excess buffer was removed by immersing the slide briefly in 200 mL deionised water, after which slides were then dried by centrifugation (240 × g for 2 min) at room temperature. Slides were then stored at room temperature and scanned the same day at 10 µm resolution using an Agilent G2505C fluorescence microarray laser scanner.

**Fig. 2:**
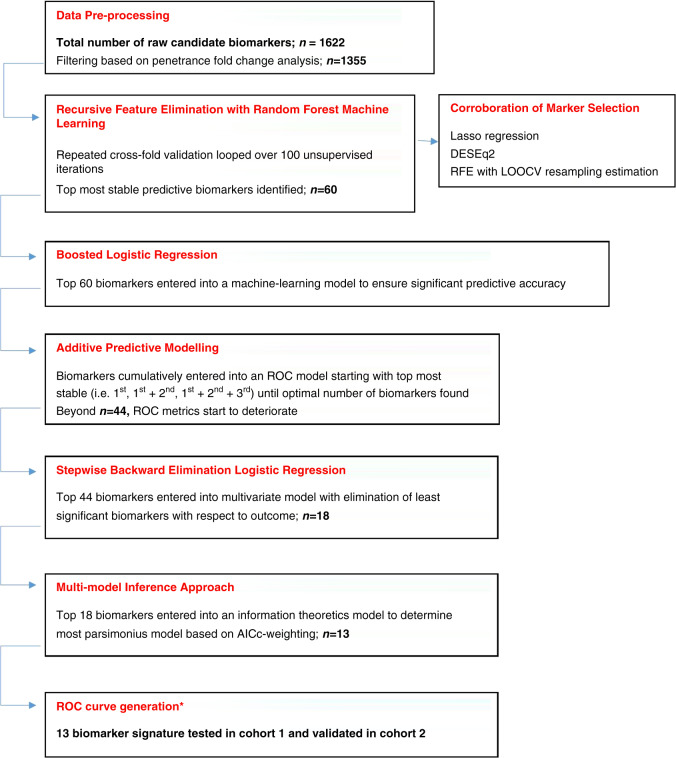
Data analysis algorithm illustrating steps in raw data handling to applied machine-learning processes and generation and testing of a final biomarker panel in the validation cohort. Throughout the algorithm, we indicate the number of biomarkers that are successively eliminated based on variable importance and stability in the model. At each iterative step, the number of biomarkers is displayed indicating successive removal and refinement of the model.

An outline of the bioinformatics analysis algorithm is shown in Fig. 2.

### Data pre-processing

Scanned images were pre-processed and quality control checks were performed on the generated data using the Sengenics internal pipeline [9]. Composite normalisation of the data was done subsequently by using both quantile- and intensity-based modules on the Cy3-labelled biotinylated BSA-positive control probes as reported by Duarte et al. [10]. AAb binding towards specific proteins was presented as relative fluorescent intensities (RFUs) and used as inputs for downstream analysis.

### Penetrance fold-change analysis

The penetrance fold-change (pFC) analysis compares both the frequency and strength of AAb signals with the intention of identifying biomarkers that are highly elevated in survivors. To achieve this, individual FCs of survivors and non-survivors were estimated using the equation below:

$${{{{{{{\mathrm{IFC}}}}}}}}_{\left( {{{{{{{{\mathrm{protein}}}}}}}}\,{{{{{{{\mathrm{A}}}}}}}},\,{{{{{{{\mathrm{sample}}}}}}}}\,{{{{{{{\mathrm{X}}}}}}}}} \right)} = {{{{{{{\mathrm{RFU}}}}}}}}_{\left( {{{{{{{{\mathrm{protein}}}}}}}}\,{{{{{{{\mathrm{A}}}}}}}},\,{{{{{{{\mathrm{sample}}}}}}}}\,{{{{{{{\mathrm{X}}}}}}}}} \right)}/{\upmu}\,{{{{{{{\mathrm{RFU}}}}}}}}_{({{{{{{{\mathrm{protein}}}}}}}}\,{{{{{{{\mathrm{A}}}}}}}},\,{{{{{{{\mathrm{control}}}}}}}}\,{{{{{{{\mathrm{group}}}}}}}})}$$

Protein A represents each protein in the Immunome array and X represents every sample assayed in the microarray platform. The mean RFU value for each protein in the control group was used as a background threshold.

For both the survivor and non-survivor groups, respectively, pFC values for each group were obtained by calculating the mean IFC of patients who pass the IFC threshold of ≥2. The penetrance frequencies were then calculated by estimating the number of patients (in each group) who has an IFC ≥2 [11]. Biomarkers were further filtered based on the criteria of (i) pFC of survivors ≥2, (ii) % penetrance frequency of survivors ≥10% and (iii) penetrance frequency of non-survivors ≤10%.

### Selection of biomarker panel

A combination of feature selection and machine-learning methodologies were used to determine the optimal number of biomarkers that were able to provide the best stratification between survivors and non-survivors [12]. For feature selection, univariate statistical tests, random forest importance and mutual information metrics were used as filter methods to rank biomarkers (the full list of filter functions are listed in Supplementary Methods (S2)). Given the degree of multi-collinearity between the biomarkers, Recursive-feature elimination (RFE) with random forest modelling was applied to the dataset, looping across 100 unsupervised iterations using random seeds for marker reliability. The topmost stable biomarkers were used to generate biomarker panels by additively selecting the top-ranking biomarkers (top 3.75% of biomarkers, *n* = 60) in a cumulative fashion, starting with the most stable biomarker from the RFE set (i.e. 1st, 1st + 2nd, 1st + 2nd + 3rd etc). Receiver operating characteristic (ROC) metrics were determined for each additive model and the top-performing combination was taken forward as input to machine-learning models. Any further addition of biomarkers did not lead to significant improvements in model performance, but only further increases in computational time. To determine the biomarker panel performance, ROC, sensitivity and specificity were evaluated and the biomarker panel with the best sensitivity and specificity was deemed the optimal panel to stratify between survivors and non-survivors. For this analysis, Boosted Logistic Regression was performed under default settings using accuracy estimation methods, repeated cross-fold validation and leave-one-out cross-validation [13].

### Model selection

To corroborate marker selection from the RFE algorithm, we used lasso regression with repeated tenfold cross-validation in the training set. This was applied using the R package glmnet. We set the elastic-net penalty, *α*, that bridges the gap between lasso (*α* = 1, the default) and ridge regression (*α* = 0), to 0.9 for numerical stability [14]. Furthermore, we processed proteomics data using DESeq2 (v.4.0.2) software to identify differentially expressed proteins between survivors and non-survivors. A cut-off of gene expression FC of ≥2 or ≤0.5 and a false discovery rate *q* ≤ 0.05 was applied to select the most differentially expressed proteins.

### Akaike information criterion

We adopted a model averaging approach using the Akaike information criterion (AIC) weights [10, 15] in order to estimate the in-sample prediction error and thereby the relative quality of the statistical models for a given set of data. We used an information-theoretic approach to calculate the AIC for each model permutation within the top-ranking biomarkers using the glmulti and MuMIn packages in order to determine the most parsimonious model with the greatest explanatory predictive power. The AIC is a measure of how well a model fits the data relative to the other possible models given the data analysed and favours fewer parameters [16]. The model with the lowest AIC is the best model approximating the outcome of interest. AIC can be expressed as:

$${{{{{{{\mathrm{AIC}}}}}}}} = - 2\left( {{{{{{{{\mathrm{log}}}}}}{\mbox{-}}{{{{{\mathrm{likelihood}}}}}}}}} \right) + 2{{{K}}},$$

where *K* is the number of model parameters and log-likelihood is a measure of model fit. In this study, as *n*/*K* ≤ 60 for sample size *n* and the model with the largest value of *K*, we used the second-order bias correction version of the AIC (AICc):

$${{{{{{{\mathrm{AICc}}}}}}}} = - 2\left( {{{{{{{{\mathrm{log}}}}}}{\mbox{-}}{{{{{\mathrm{likelihood}}}}}}}}} \right) + 2{{{K}}} + 2{{{K}}}({{{K}}} + 1){{{n}}} - {{{K}}} - 1,$$

$${{{{{{{\mathrm{AICc}}}}}}}} = {{{{{{{\mathrm{AIC}}}}}}}} + 2{{{K}}}({{{K}}} + 1){{{n}}} - {{{K}}} - 1,$$

where *n* is the sample size, *K* the number of model parameters and log-likelihood is a measure of model fit [15, 17]. From an information-theoretic perspective, the Akaike weights for a particular model can be regarded as the probability or “weight of evidence” that the model is the best model (in a Kullback–Leibler sense of minimising the loss of information when approximating full reality by a fitted model) out of all of the models considered/fitted based on the available dataset [15, 16].

## Results

### Identification of predictive biomarkers (Fig. 2)

Initial data processing involved filtering according to the pFC analysis in order to avoid biasing subsequent model generation. One thousand three hundred and fifty-five biomarkers remained, which were taken forward into the deeper analysis. The biomarkers, which appeared most frequently with the highest importance values across 100 randomly seeded iterations, are listed in Supplementary Table (S3). Corroborative regression and genomics analysis methods were performed and indicate the biomarkers, which were common to all analytical techniques. Overall, 60 biomarkers (RFE set) were identified as the most stable with no improvement in predictive performance beyond this number.

### Additive predictive modelling

The RFE set of biomarkers was used to generate biomarker panels by additively selecting the top-ranking biomarkers in a cumulative fashion. These inputs were used to determine the ROC metrics at each additive iteration for cohort 1, displayed in Supplementary Graph (S4). An upward linear trend in all three parameters (area under the curve (AUC), sensitivity, specificity) was noted as more biomarkers were added. This progressive increase peaked at 44 cumulative biomarkers (AUC 0.975; sensitivity 87%; specificity 98.5%). Beyond this, the predictive metrics become rather unstable and less uniform, hence the decision to proceed with the top 44 biomarkers for deeper analysis.

### Multi-model inference approach

Given that a 60-biomarker diagnostic scoring system would be cumbersome and impractical, we utilised an information-theoretic approach to determine the biomarker combination with the highest diagnostic potential in the most parsimonious model. We employed the AICc method in order to estimate the “goodness of fit” of statistical models and thereby compare multiple models with one another. The AICc avoids overfitting the model in smaller sample sizes. Based on the cumulative ROC analysis, we proceeded with the top 44 biomarkers in this downstream analysis. Following stepwise backward elimination of these markers in a multivariate logistic regression model, with survivorship as the dependent variable, 18 biomarkers were determined to be the most significant and were therefore used in the multi-model inference analysis. Any further addition of more biomarkers did not lead to significant improvements in model performance, but did contribute to significant increases in computational time.

### Assessing model performance

**Fig. 3:**
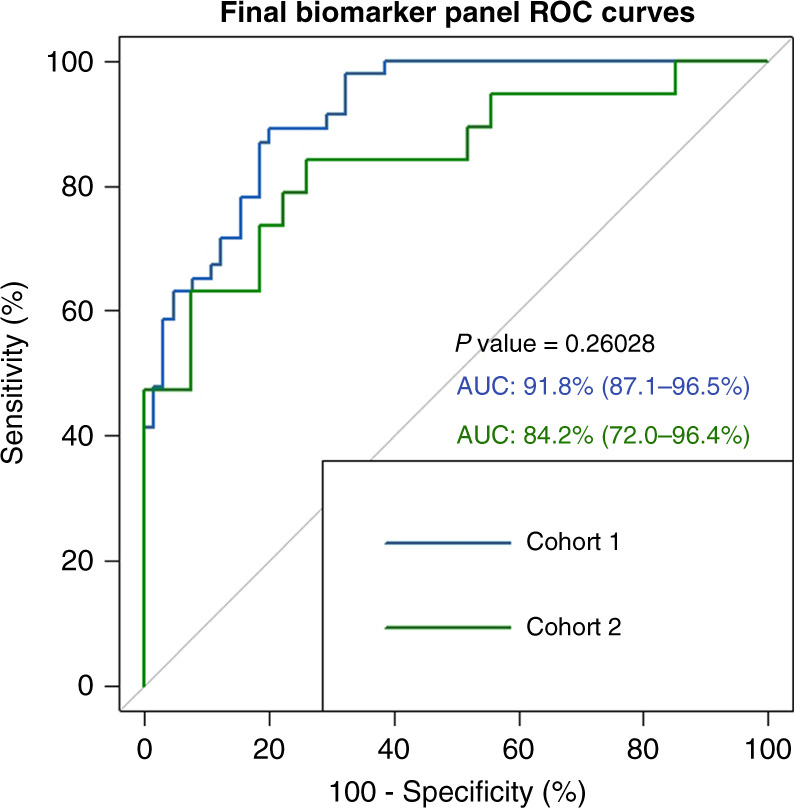
ROC curves for Biomarker Panel A in both Cohorts. ROC curves demonstrating the performance of panel (**a**) (13 biomarkers) in both cohort 1 (test) and cohort 2 (validation). *P* value indicates no significant difference in the performance of this model between cohorts. AUC 95% confidence intervals are displayed within brackets.

**Fig. 4:**
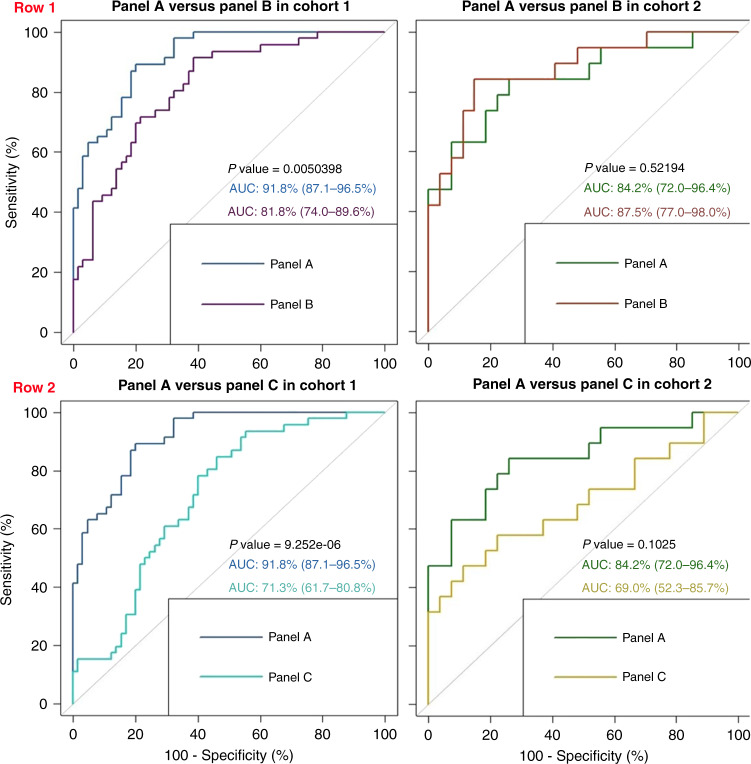
Comparative ROC curves for Biomarker Panel combinations in both Cohorts. ROC curves demonstrating performance of panel (**a**) (13 biomarkers) and panel (**b**) (16 CTAG biomarkers from RFE set) in cohort 1 (test) (row 1, left panel) and cohort 2 (validation) (row 1, right panel. AUC confidence intervals are displayed within brackets. *P* values indicate significant differences in performance in cohort 1 but not cohort 2. ROC curves demonstrating performance of panel (**a**) (13 biomarkers) and panel (**c**) (6 CTAG biomarkers from panel (**a**)) in cohort 1 (test) (row 2, left panel) and cohort 2 (validation) (row 2, right panel). AUC confidence intervals are displayed within brackets. *P* values indicate significant differences in performance in cohort 1 but not cohort 2.

Panel a, the most parsimonious and best-performing model, comprised 13 biomarkers—SPATA19, TSPY3, GLS2, TCEA2, TSGA10, HMGN5, LUZP4, HDAC4, SPACA3, IMPDH1, TXN2, TFG and PPP2R1A (Supplementary Table (S5)). ROC metrics for each individual candidate biomarker are found in Supplementary Table (S6), along with association with clinico-pathological correlates (Supplementary Figure S7). This refined model was assessed in cohort 1 (AUC 0.918, sensitivity 89.1%, specificity 80.1%) and validated in the independent cohort 2 (AUC 0.842, sensitivity 84.2%, specificity 74.1%) (Fig. 3). There was no significant difference in the ROC metrics between the two cohorts, indicating good performance in the validation cohort. We noted a preponderance of bona fide cancer testis antigens (CTAGs) in the RFE biomarker set (16/60 (26.7%)). We thus elected to explore two further CTAG specific panels in order to determine the prognostic relevance of these highly conserved proteins in NSCLC. We refer to the final biomarker panel we defined as panel a (13 biomarkers). Panel b refers to the CTAGs extracted from the RFE set (16 biomarkers) and panel c refers to the CTAGs extracted from panel a (6 biomarkers). The strong CTAG presence in panel a comprises six proteins antigens, SPATA19, SPACA3, TSPY3, TCEA2, TSGA10 and LUZP4, all with established pro-tumourigenic roles in different cancers under certain conditions (Supplementary Table (S5)). CTAGs trigger unprompted humoral immunity and immune responses in malignancies, altering tumour cell physiology and neoplastic behaviours. Their limited expression in normal somatic tissues coupled with recurrent up-regulation in epithelial carcinomas makes them highly attractive biomarker and vaccine targets. We explored the performance of all three panels in cohorts 1 and 2 (Fig. 4). Panel a performed significantly better in cohort 1 (test) than both panels b and c (CTAG panels). However, in cohort 2 (validation), the differences between panel a and the CTAG panels (b and c) was not significant. Panel b (16 CTAG panels) outperformed panel a in cohort 2 (AUC 0.875 versus 0.842, *p* = NS), but panel c underperformed compared to panel a in cohort 2 (AUC 0.69 versus 0.842, *p* = NS). The increased predictive performance of panel b (16 CTAG panels) reaffirms the importance of CTAGs in discriminating between survivorship in lung cancer. In spite of CTAG preponderance, these data show that the non-CTAG antigens in panel a, which are critical mediators of Wnt signalling and phosphatase activity, are clearly biologically important in their ability to prognosticate in lung cancer.

### Survival analysis

**Fig. 5:**
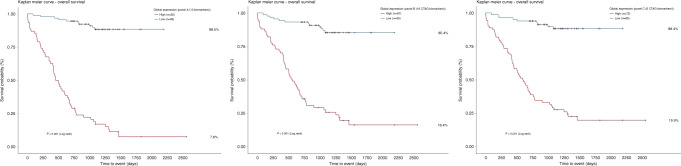
Kaplan-Meier Survival Curves. Kaplan–Meier survival analysis for panel (**a**) (13 biomarkers), panel (**b**) (16 CTAG biomarkers from RFE set) and panel (**c**) (6 CTAG biomarkers from panel (**a**)). All curves demonstrate significantly worse outcomes in patients with high global expression of each biomarker signature. The number of patients in each expression group is shown within brackets in the legend for each Kaplan–Meier plot. Percentage survival out to 5 years is displayed to the right-hand side for each expression group in each plot. The median survival for high expressers of panels (**a**, **b** and **c**) is 479, 558 and 600.5 days, respectively.

**Fig. 6:**
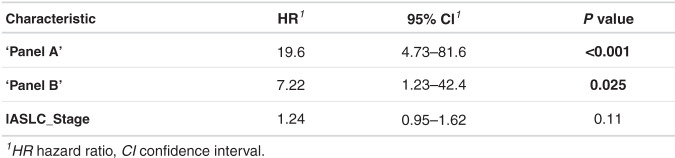
Multivariate Cox proportional hazards modelling in entire NSCLC cohort (*n* = 157). Stepwise backward elimination was employed to remove the least significant independent predictors.

Further interrogation of these signatures was carried out by generating a continuous risk score for every individual on the basis of model coefficients. The resultant predicted risk scores from cohort 1 (training) were divided using optimal cut-off points determined through ROC analysis in order to further dichotomise the patient cohorts as “high expressors” and “low expressors”. We performed this for all three panels (a–c), the scores being inferred directly from the biomarker signal intensities. Using these individual risk scores, we carried out survival analyses (Fig. 5) and multivariate Cox proportional hazards modelling (Fig. 6) in the entire NSCLC cohort. Patient age, gender, histology, nodal status, IASLC stage, lymphovascular invasion and whether patients underwent adjuvant chemotherapy were all predictors that were entered into the model alongside all the panel scores. All panels were able to effectively dichotomise between survivor statuses in our cohort, with high expression conferring a significantly worse outcome (*p* < 0.001), reaffirming findings from the ROC analysis. Five-year survival in high expressers of panels a, b and c was 7.6%, 16.4% and 19.9%, respectively, and high expressers of panel a had a median survival of just under 16 months, which for early-stage resected lung cancer is very low. On multivariate testing, only panels a and b were deemed significant independent predictors of survival, HR 19.6 and 7.22, respectively (*p* < 0.05). IASLC stage was still deemed an independent predictor of outcome albeit not significant (HR 1.24, *p* = 0.11). Panel c was deemed a significant independent predictor of outcome only when entered into a multivariate model without panels a and b. This reaffirmed the findings that the CTAGs alone from panel a were not sufficiently predictive enough when compared with panels a and b, but are still significant predictors independent of age, gender, IASLC stage, lymphovascular invasion, histology, nodal status and whether patients underwent adjuvant chemotherapy.

We performed multivariate analyses in various subgroups according to gender, histology ISALC stage and adjuvant therapy status to explore the relevance of panel a in relation to specific clinico-pathological factors. In all subgroups, panel a was the most significant independent predictor of outcome (Supplementary Table (S8)).

## Discussion

Results from the NLST and European NELSON trials were strongly supportive of lung cancer screening [18, 19]. Widespread use of CT coronary angiography to assess inpatient chest pain as well as the use of whole-body CT use in the assessment of Trans-catheter valve intervention results in a high detection rate of incidental findings, a large proportion of which are lung malignancies. The combination of screening strategies and increased use of CT scanning for non-cancer-related conditions will result in a surge in the detection rate of early-stage lung cancers and therefore an increased surgical resection rate. Early-stage lung cancers confer a multitude of outcomes ranging from indolent disease with high post-operative disease-free survival rates at 5 years to highly aggressive disease with relapse in the first 12 months post resection.

Current prognostic biomarkers for early-stage lung cancers have been described but are limited in their utility owing to the lack of proper validation and lack of adequate sensitivity and/or specificity. The key points in evaluating biomarker studies in early-stage lung cancer include well-defined objectives and study populations, robust specimen storage and use, and the use of a clinically applicable assay that is validated in an independent cohort of patients. Critical appraisal of published prognostic signatures in early-stage lung cancers found that adherence to these criteria was poor with overt flaws in study design. Subramanian and Simonet al published a set of guidelines to inform prognostic biomarker studies in lung cancer, and although all the studies pertain to gene expression microarray data, we have adhered to these criteria as closely as possible [20]. We validated our signature in one completely independent dataset, which is a feature that is lacking in many prognostic signatures in early-stage lung cancer [20]. A 14-gene, quantitative real-time PCR-derived expression signature was previously validated in two independent large stage I, non-squamous NSCLC datasets, which demonstrated the robustness of the statistical design. This signature showed poor survival in high-risk patients based on gene expression in both validation cohorts, and although the AUC values were significantly higher for this signature than standard NCCN risk criteria in both validation sets, the absolute values were still relatively low (0.60 and 0.61), compared with the AUC values from our panels a and b (AUC 0.842 and 0.875) [21]. Furthermore, this study did not assess the therapeutic relevance of the genes identified in the final signature.

Historically, the majority of AAb-based biomarker research, including that in NSCLC, has concentrated on the diagnosis of disease states or early detection of cancers as opposed to trying to map the course of disease post treatment [22]. Sensitivities and specificities of biomarker panels for lung cancer detection have ranged from 0 to 92.2% and 79.5 to 92.2%, respectively [23].

Circulating proteins have also been investigated as prognostic biomarkers in early-stage lung cancer, the most common of which are CEA and CYFRA 21-1. The largest study exploring the role of CEA found that elevated pre-operative levels conferred poor 5-year survival [24].

Given the complexity and multi-factorial nature of the anti-tumour immune response and tumour immune evasion mechanisms in cancers that are not solely reliant on single oncogenic drivers, combination biomarker signatures are more valuable [23]. None of the prognostic studies offered any predictive assessment of their panels, but instead used hazard ratios (measures of association, not predictive power) with no separate test/validation.

Three broad categories of genes comprised our final panel (panel a), namely CTAG expression, Wnt signalling protein aberrancy and serine/threonine protein phosphatase deregulation. CTAGs are united by their role in embryonic development and restriction of expression to male germ cells. Ectopic re-expression of these antigens has been seen in a variety of somatic solid tumours and in triple-negative breast cancers, high expression is correlated with worse survival in multivariate analysis (HR 2.02, 95% confidence interval 1.27–3.20; *p* = 0.003) [25]. Ectopic gene signatures of normally silenced CTAG genes that are expressed in cancer associated with a highly aggressive lung cancer phenotype and independently predicted poor outcome [26]. We identified 16 CTAGs (27%) in our RFE set (S3) as being highly discriminatory for survivorship in this distinct cohort of NSCLC patients (SPATA19, SPACA3, TSGA10, TSPY3, LUZP4, TCEA2, CTNNA2, MAGEB2, SPO11, MAGEB4, MAEL, CSAG1, MAGEB5, COX6B2, GAGE2 and TSSK6). This CTAG only model displayed high predictive power in the validation cohort (AUC 0.875, sensitivity 84.2%) and was a significant independent predictor of poor outcomes. Clonal and subclonal CTAG expansion is generally uniform in tumour cells with variations in behaviour tightly regulated by epigenetic alterations [27].

Aberrant activation of the Wnt/β-catenin signalling pathway is causally linked to cancer recurrence, immune evasion and metastasis. A number of the identified tumour-associated antigens are known to signal via this cascade, HMGN5, TFG, MAEL, SOX15 and Dicckopf-1, the latter of which has been investigated in numerous other biomarker signatures [28–30] and like TFG interacts via the Wnt co-receptor LRP6 [31]. Proteins such as MAEL and PTK7 both signal via this cascade and the expression was significantly associated with poor outcomes in our cohort. MAEL, also a CTAG, has been shown to be critical for cancer cell survival and is over-expressed in the bladder and gastric cancers [32]. Functional experiments have determined that MAEL protein exerts its oncogenic dominance through degradation of the protein phosphatase ILKAP [33, 34]. Mutating or silencing of phosphatase activity is a well-known tumour escape mechanism [35, 36] and is the third core component of our identified biomarker panel. MAEL provides a unique link between all three biological pathways. These molecules provide unique therapeutic targets as demonstrated by an antibody–drug conjugate against the Wnt signalling PTK7 tyrosine kinase molecule, which elicited potent anti-tumour activity in low-passage patient-derived solid tumour xenograft models [37]. In solid tumours like NSCLC, high PTK7 expression confers significantly reduced overall survival [37]. Targeting this molecule in phase I trials have just completed accrual [NCT02222922]. Multiple other agents targeting the Wnt signalling axis that have entered or completed phase I clinical trials and include Vantictumab, a monoclonal antibody against the Fzd receptor (NCT01345201, NCT02005315, NCT01957007 and NCT01973309), decoy receptors such as OMP-54F28 (NCT02069145, NCT02092363, NCT02050178 and NCT01608867) and porcupine enzyme inhibitors (NCT01351103 and NCT02521844) [28, 38].

Cellular responses to DNA damage are integral to maintaining the genome and preventing cancer progression; serine–threonine phosphatases like protein phosphatase 2 play a key role in the DNA damage response through the regulation of important cell cycle proteins and tumour suppressor genes such as *ATM*, *Chk1*, *Chk2*, *p53* and *BRCA1* [36]. Cancer cells tend to evade the activation of DNA repair pathways through copy number alterations of Ser/Thr phosphatases, missense mutations and increased mutant gene expression. Identifying aberrancy of these important proteins and utilising early antigen expression is key to disease surveillance and therapeutics. Following the exploitation of BCR/ABL kinase inhibition in chronic myeloid leukaemia, efforts have been made to explore PP2A phosphatase reactivation/inhibition in anti-tumour therapy. PPP2R1A dysregulation was individually a significant independent predictor of poor survival in both cohorts; belonging to the PP2A enzyme family, these complexes exert control over oncogenic signalling pathways (MEK/ERK and Srk-Jnk) and over collateral resistance phosphorylation pathways. Their inhibition in a KRAS-mutant human lung cancer cell line resulted in improved responses with MEK inhibitors [39]. Mutations of PPP2R1A significantly enhance cancer cell migration in endometrial and ovarian carcinomas [40], whereas allosteric activation of this wild-type complex induces cell cycle arrest with broad anti-tumour activity [35]. Notably, over-expression and mutation of the target antigen are two of the classical mechanisms for AAb production, with CD4^+^ T-helper cells specific to mutated neoepitopes being able to drive the expansion of a set of antigen-specific B cells, resulting in the secretion of polyclonal AAbs that are able to recognise both mutated and wild-type forms of the antigen [41]. Thus, our observation that anti-PPP2R1A AAbs are independent predictors of poor survival is consistent with the known aberrant function of PPP2R1A in oncogenesis, albeit our data does not allow us to yet distinguish between the two possible molecular origins of the AAbs. Current phase 2 trials in recurrent glioblastoma (NCT03027388) are investigating the role of PP2A inhibitor, LB100.

Whilst the aim of this study was to identify a highly prognostic panel for surgically resectable lung cancer, the biology of the final markers suggests a line of sight to the clinic in terms of adjuvant therapies, in which high expression of our prognostic biomarker might indicate a very poor outcome group of patients whose survival might be improved by targeting some of the proteins that the AAbs have formed against particularly the CTAGs. The highly restricted expression patterns of CTAGs in normal tissues and ectopic expression in tumour types makes them highly sought after as targets for cancer vaccines [31]. The Lipo-MERIT trial demonstrated strong CD4^+^ and CD8^+^ T cell induction along with durable objective clinical benefit in unresectable melanoma patients treated with a poly-antigenic liposomal RNA vaccine with or without combination with anti-PD1 checkpoint blockade therapy [32]. The RNA vaccine targeted four main CTAGs: NY-ESO-1, MAGEA3, TPTE and Tyrosinase [32]. In our dataset, low CTAG expressers (Fig. 5) had good outcomes, with 85.4% 5-year overall survival (*p* < 0.001); targeting this group is unlikely, therefore, to be of benefit; however, high expressers who suffer poor outcomes post resection may well be suitable for a CTAG-based polyepitopic RNA vaccine as an adjunct to standard adjuvant chemotherapy in order to further eliminate micro-metastatic deposits and cells with a high biological propensity for aggressive disease.

## Limitations and further work

Overall study limitations include the retrospective design and heterogeneity of the study population, which can introduce selection bias. This allied with the clinical diversity of the population may mean the results are less easy interpretable. However, we sought to mitigate against this using our robust random machine-learning-based approach. Despite good performance, the panel identified should also be employed to determine clinical utility in larger independent NSCLC cohorts with the parsimonious panel as well as in other cancers to ascertain if the signature is disease-specific.

There are additional intrinsic limitations related to the identification of proteins markers in biological fluids, in particular the expression of AAb against highly conserved intracellular tumour markers or in immuno-privileged sites. The host immune response against cancer antigens is complex and tends to direct itself towards the most immunogenic epitopes. It is known that autoantigens that are modified before or during the course of tumour formation and progression in cancer can stimulate the immune response in patients when they are released from tumour cells and that immune responses have been observed to be responsible for tumour growth promotion, but also prevention in a process called immuno-editing [42]. Further underscoring this, most of the seroreactive biomarkers in the RFE set (*n* = 60) are intracellular antigens (52/60) interacting with membrane and non-membrane-bound organelles such as ribosomes (4/60), with the majority residing within the nucleus (37/60), a usually immuno-privileged site. This pattern has been observed in AAb studies in melanoma [42]. Despite this, AAbs generated against autologous nuclear antigens are frequently found in cancer patient sera [43]. Nuclear antigens, however, do not undergo antigen presentation during the negative selection of self-reactive lymphocytes largely because of their intrinsic proteolytic instability, which affects the binding kinetics with major histocompatibility complex class II receptors. Exposure of the nuclear antigens to one’s immune system and the resultant generation of AAbs is, therefore, thought to occur following tumour cell death and release of the intracellular contents into the circulation, although altered cellular localisation [44], or shedding in exosomes, in transformed cells may also play a role. The understanding of these key points might help to clarify the response of our body against cancer autoantigens in a patient-specific manner, but further clinical validation is needed in order to extend the use of these 13 biomarkers in early detection and mapping the prognosis of cancer.

## Supplementary information

S1

S2

S3

S4

S5

S6

S7

S8

List of Supplementary Material

REMARK checklist

## Data Availability

The datasets generated during and/or analysed during the current study are available from the corresponding author on reasonable request.

## Code availability

The authors declare that the code for reproducibility of data are publicly available or will be available upon request.
