# Supplementary material for: A highly predictive autoantibody-based biomarker panel for prognosis in early-stage NSCLC with potential therapeutic implications
Source: Br J Cancer. 2021 Nov 2;126(2):238–46. doi: 10.1038/s41416-021-01572-x (PMC8770460; doi:10.1038/s41416-021-01572-x)
Supplement: Supplementary file 1 — S1 [file 41416_2021_1572_MOESM1_ESM.docx]

**Supplementary Table (S1) – Clinicopathological Characteristics of the Study Cohorts**
