# Supplementary material for: A highly predictive autoantibody-based biomarker panel for prognosis in early-stage NSCLC with potential therapeutic implications
Source: Br J Cancer. 2021 Nov 2;126(2):238–46. doi: 10.1038/s41416-021-01572-x (PMC8770460; doi:10.1038/s41416-021-01572-x)
Supplement: Supplementary file 2 — S2 [file 41416_2021_1572_MOESM2_ESM.docx]

**Supplemental Methods (S2)**

Additional Statistical Analyses

All other statistical analyses were done using the RFU values of 1600+ proteins using the R platform. ROC analyses were performed using the package OptimalCutpoints (1) and plotted using ggplot2 (2). Survival analyses were performed using survminer package (3). Machine learning analyses were performed using the mlr (4)*,* party (5)*,* ranger (6)*,* randomForest (7) and praznik (8) and caret (9) package. Power calculations were performed using the samplesize and sizepower packages (9–11). Data presentation in table format was implemented using the gtsummary package (12).

List of filter functions applied to determine the optimal biomarker panel

| **Name** | **Test** | **R package** |
| --- | --- | --- |
| anova.test | univariate statistical test | mlr |
| auc | univariate statistical test | mlr |
| kruskal.test | univariate statistical test | mlr |
| party_cforest.importance | random forest importance | party |
| praznik_CMIM | mutual information | praznik |
| praznik_JMI | mutual information | praznik |
| praznik_JMIM | mutual information | praznik |
| praznik_MIM | mutual information | praznik |
| praznik_MRMR | mutual information | praznik |
| praznik_NJMIM | mutual information | praznik |
| praznik_DISR | mutual information | praznik |
| randomForest_importance | random forest importance | randomForest |
| ranger_permutation | random forest importance | ranger |
| ranger_impurity | random forest importance | ranger |
| variance | feature variance | mlr |

Supplemental Methods References

1. López-Ratón M, Rodríguez-Álvarez MX, Suárez CC, Sampedro FG. **OptimalCutpoints** : An *R* Package for Selecting Optimal Cutpoints in Diagnostic Tests. J Stat Softw. 2014;61(8).

2. Wickham H. ggplot2. Cham: Springer International Publishing; 2016.

3. Kassambara A, Kosinski M, Biecek P, others. survminer: Drawing Survival Curves using’ggplot2’. R Package Version 03. 2017;1.

4. Bischl B, Lang M, Kotthoff L, Schiffner J, Richter J, Studerus E, et al. Mlr: Machine Learning in R. J Mach Learn Res. 2016 Jan;17(1):5938–5942.

5. Strobl C, Boulesteix AL, Kneib T, Augustin T, Zeileis A. Conditional variable importance for random forests. BMC Bioinformatics. 2008 Dec 11;9(1).

6. Wright MN, Ziegler A. Ranger: A fast implementation of random forests for high dimensional data in C++ and R. J Stat Softw. 2017;77(1).

7. Breiman L. Random forests. Mach Learn. 2001;45(1):5–32.

8. Kursa MB. praznik: Tools for Information-Based Feature Selection. 2020.

9. Kuhn M. Building predictive models in R using the caret package. J Stat Softw. 2008;28(5):1–26.

10. Qiu W. Sample Size and Power Calculation in Microarray Studies Using the sizepower package. :8.

11. Scherer R. shearer/samplesize [Internet]. 2019 [cited 2021 Mar 6]. Available from: https://github.com/shearer/samplesize

12. Presentation-Ready Data Summary and Analytic Result Tables • gtsummary [Internet]. [cited 2021 Mar 23]. Available from: http://www.danieldsjoberg.com/gtsummary/
