# Supplementary material for: A highly predictive autoantibody-based biomarker panel for prognosis in early-stage NSCLC with potential therapeutic implications
Source: Br J Cancer. 2021 Nov 2;126(2):238–46. doi: 10.1038/s41416-021-01572-x (PMC8770460; doi:10.1038/s41416-021-01572-x)
Supplement: Supplementary file 3 — S3 [file 41416_2021_1572_MOESM3_ESM.docx]

**Supplementary Table (S3). Top most stable biomarkers as determined by recursive feature elimination**

| **Biomarker** | **Repeated k-fold**  **cross validation** | **LOOCV** | **Lasso**  **Regression** | **DESeq2** |
| --- | --- | --- | --- | --- |
| SPATA19 | 🏱 | 🏱 |  |  |
| CASP7 | 🏱 | 🏱 |  |  |
| TSPY3 | 🏱 | 🏱 |  |  |
| GLS2 | 🏱 | 🏱 | 🏱 | 🏱 |
| TCEA2 | 🏱 | 🏱 |  |  |
| CTNNA2 | 🏱 | 🏱 |  | 🏱 |
| ITPKB | 🏱 | 🏱 |  |  |
| AFF4 | 🏱 | 🏱 |  |  |
| MAGEB2 | 🏱 | 🏱 | 🏱 |  |
| C1orf174 | 🏱 | 🏱 | 🏱 |  |
| TSGA10 | 🏱 | 🏱 |  |  |
| TYRO3_int | 🏱 |  | 🏱 |  |
| DCBLD2 | 🏱 |  | 🏱 | 🏱 |
| PCLAF | 🏱 |  |  |  |
| SPO11 | 🏱 | 🏱 |  |  |
| BPIFA1 | 🏱 | 🏱 |  |  |
| MAGEB4 | 🏱 | 🏱 | 🏱 |  |
| HMGN5 | 🏱 |  | 🏱 |  |
| MAEL | 🏱 |  |  |  |
| LUZP4 | 🏱 |  | 🏱 |  |
| HDAC4 | 🏱 |  |  |  |
| SOX15 | 🏱 |  |  |  |
| HOOK1 | 🏱 |  |  |  |
| CDK16 | 🏱 |  | 🏱 |  |
| CSAG1 | 🏱 |  |  |  |
| SPACA3 | 🏱 |  |  |  |
| IMPDH1 | 🏱 |  |  |  |
| MAGEB5 | 🏱 |  |  |  |
| TXN2 | 🏱 |  | 🏱 | 🏱 |
| NFYA | 🏱 |  |  |  |
| PHF7 | 🏱 |  | 🏱 | 🏱 |
| HIST1H1C | 🏱 |  |  |  |
| IP6K1 | 🏱 |  |  |  |
| TFG | 🏱 |  |  |  |
| AIM2 | 🏱 |  |  |  |
| SGO1 | 🏱 |  |  |  |
| PYCR1 | 🏱 |  |  |  |
| FAM50B | 🏱 |  |  |  |
| HK2 | 🏱 |  |  |  |
| ERBB3_int | 🏱 |  |  |  |
| TBL1X | 🏱 |  | 🏱 |  |
| ZNF207 | 🏱 |  | 🏱 |  |
| EEF1D | 🏱 |  |  |  |
| PPP2R1A | 🏱 |  |  |  |
| MAP2K7 | 🏱 |  |  |  |
| RPL7A | 🏱 |  |  |  |
| CBLC | 🏱 |  |  |  |
| COX6B2 | 🏱 |  |  |  |
| ACTB | 🏱 |  |  |  |
| CA9 | 🏱 |  |  |  |
| FLCN | 🏱 |  |  |  |
| GAGE2 | 🏱 |  |  |  |
| ARAF | 🏱 |  |  |  |
| AK3 | 🏱 |  |  |  |
| HMG20B | 🏱 |  |  |  |
| CNN1 | 🏱 |  |  |  |
| EPAS1 | 🏱 |  |  |  |
| EAPP | 🏱 |  |  |  |
| TSSK6 | 🏱 |  |  |  |
| GRK6 | 🏱 |  |  |  |
