# Supplementary material for: A highly predictive autoantibody-based biomarker panel for prognosis in early-stage NSCLC with potential therapeutic implications
Source: Br J Cancer. 2021 Nov 2;126(2):238–46. doi: 10.1038/s41416-021-01572-x (PMC8770460; doi:10.1038/s41416-021-01572-x)
Supplement: Supplementary file 4 — S4 [file 41416_2021_1572_MOESM4_ESM.docx]

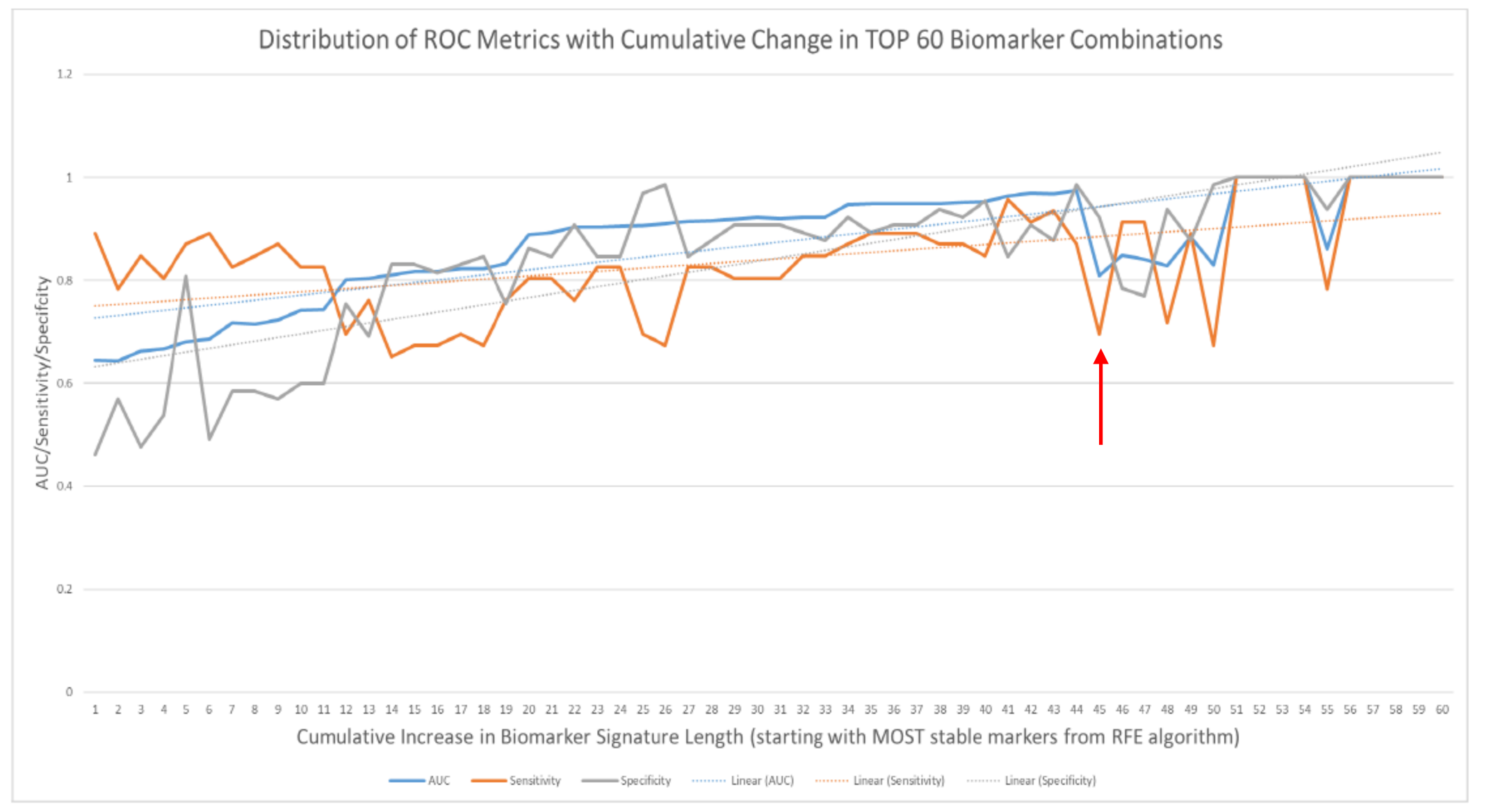
**Supplementary Graph (S4):** ROC metrics displayed for additive modelling of biomarkers in the RFE set (n=60). There is a progressive linear increase in AUC (blue), sensitivity (orange) and specificity (grey) with each cumulative addition of biomarkers. The solid lines show the interval change in each metric with each biomarker addition and the dashed lines represent the overall trend. There is a progressive improvement in all parameters up to 44 biomarkers, beyond which, there is a decay in performance for each metric as shown by the sharp negative deflection (red arrow). The performance of the model deteriorated beyond 44 cumulative biomarkers indicating no added value.
