# Supplementary material for: A highly predictive autoantibody-based biomarker panel for prognosis in early-stage NSCLC with potential therapeutic implications
Source: Br J Cancer. 2021 Nov 2;126(2):238–46. doi: 10.1038/s41416-021-01572-x (PMC8770460; doi:10.1038/s41416-021-01572-x)
Supplement: Supplementary file 5 — S5 [file 41416_2021_1572_MOESM5_ESM.docx]

**Supplementary Table (S5). A comprehensive overview of the 13 final biomarkers in Panel A**

| **Biomarker** | **Name** | **Biological Significance** | **Role in Neoplasia (TSG or Oncogene)** | **Link to cancers** | **Reference** |
| --- | --- | --- | --- | --- | --- |
| SPATA19🟀 | Spermatogenesis-associated protein 19 | Exclusively expressed in the testis, participates in cellular differentiation and spermatogenesis. Plays a role in the maintenance of the normal mitochondrial sheath and sperm motility. | Oncogene | Significantly elevated expression in prostate and basal cell carcinoma. High expression levels correlated with biochemical recurrence in prostate cancer (p=0.02). | (1–4) |
| TSPY3🟀 | Cancer Testis Antigen 78/Testis Specific Protein Y-Linked 3 | Role in gonadal differentiation and spermatogenesis. | Unclear | No known link as yet. | (5–11) |
| GLS2 | Glutaminase 2 | An intranuclear glutaminase that multi-localises to the nucleus and mitochondria. Plays an important role in the regulation of glutamine catabolism. Promotes mitochondrial respiration and increases ATP generation in cells by catalyzing the synthesis of glutamate and alpha-ketoglutarate. Increases cellular anti-oxidant function via NADH and glutathione production. | Unclear but data favours tumour suppressor role | Upregulation of this protein in cancer cells results in cell cycle arrest at the G2/M phase thus halting proliferation. Moreover GLS2 is a p53 direct target gene and negatively regualted the PI3K/AKT signalling pathway thus displaying tumour suppression activity in human hepatocellular carcinoma. Hypermethylation of the GLS2 promotor contributes to decreased expression in this cancer type and in glioblastoma independent of p53 inactivation. Different GLS2 isoforms may be responsible for poor outcome in breast cancers where it has shown to be pro-tumourigenic. In glioblastoma, concurrent silencing of GLS2 in cell lines as well as GLS2 gene overexpression cooperate to decrease proliferation and glioblastoma cell viability. |  |
| TCEA2🟀 | Transcription Elongation Factor A2/TFIIS/Testis-Specific SII gene | Intranuclear expressed protein which functions as an S2 class transcription elongation factor, responsible for releasing RNA polymerase II complexes from transcriptional arrest past template-encoding arresting sites. Member of the TFIIS family of elongation factors, TCEA2 originally identified as a testis/ovary specific gene. | Oncogene | Overexpressed on chromosome arm 20q with copy number gain in cervical cancer. TCEA2 has been shown to interact with BRCA1 at sites of UV-induced transcription associated damage in breast and ovarian cancer, with known amplification in 11 different tumour types. Targeting multiple steps in mutated repair/response pathways may evolve as part of a rational therapeutic strategy for these cancers. | (12–14) |
| TSGA10🟀 | Testis-specific gene protein 10/Cancer Testis Antigen 79 | Role in gonadal differentiation and spermatogenesis. When over-expressed, prevents nuclear localisation by HIF1-alpha. | Unclear but data favours tumour suppressor role | In vitro HUVEC cell lines derived from nasopharyngeal carcinoma have demonstrated an anti-angiogenic role for TSGA10. Over-expression in breast cancer MCF-7 cells decreased metastatic and metabolic activity of said cells through HIF-1alpha inhibition. High expression of TSGA10 in bladder cancer post-resection specimens correlated with poorer overall survival however in bladder cancer lines, high expression was associated with increased apoptosis, reduced cancer cell migration and prevention of epithelial cell transformation. | (15–18) |
| HMGN5 | High Mobility Group Nucleosome Binding Domain 5/NSBP1 | Encodes a nuclear protein with similarities to the high mobility group proteins, HMG14 and HMG17, which suggests that this protein may function as a nucleosomal binding and transcriptional activating protein. Preferentially binds to euchromatin and modulates cellular transcription by counteracting linker histone-mediated chromatin compaction. Chromatin decompaction in this manner has been shown to decrease the elastiscity and rigidity of nuclei in cultured cells. | Oncogene | HMGN5 is highly expressed in bladder cancer in murine models, resected human transitional cell carcinomas of the bladder and in vitro cell lines. In murine models, it has been shown to interact with HSP27 and promote epithelial cell transition via IL-6 signalling. HMGN5 knockdown via siRNA suppresses cellular progression in glioblastoma cell lines via AKT/MAPK signalling, and a similar effect has been demonstrated in meningioma, oesophageal squamous cell carcinoma and lung cancer cell lines. HMGN5 signalling via the Wnt/B-catenin pathway is one of the key processes that mediate cellular progression and invasion in pancreatic ductal adenocarcinomas. | (19–27) |
| LUZP4🟀 | Leucine Zipper Protein 4/Cancer Testis Antigen 28 | First defined as a cancer testis antigen; RNA binding protein that interacts with nuclear RNA export factor NXF1. | Unclear but data favours oncogenic role | Restricted expression in normal non-disease cell lines but highly expressed in melanoma and multiple myeloma cell lines where it is required for growth. Identified to a high degree in breast cancer networks. This gene is silenced in normal tissues except for testis but frequently activated in human neoplasms (lung, breast, ovarian, glioma, and melanoma) where it remains intranuclear and partakes in transcriptional activity. | (28–30) |
| HDAC4 | Histone Deacetylase 4 | Responsible for the deacetylation of lysine residues on the N-terminal part of the core histones (H2A, H2B, H3 and H4). Histone deacetylation gives a tag for epigenetic repression and plays an important role in transcriptional regulation, cell cycle progression and developmental events. This protein does not bind DNA directly, but through transcription factors MEF2C and MEF2D. | Unclear but data favours oncogenic role | Ovarian cancer frequently acquires resistance to platinum chemotherapy, representing a major challenge for improving patient survival. Analysis of the paired tumor biopsies taken before and after development of clinical platinum resistance showed significantly increased HDAC4 expression in resistant tumors. Higher expression of HDAC4 has been described in breast, bladder and hepatocellular carcinomas where it is critical to cell growth. In vitro cellular inhibition of HDAC4 in lung cancer cell lines reduced proliferation. However RT-PCR analysis of resected colon and lung carcinoma specimens revealed significantly reduced expression of HDAC4 and other p53 related tumour suppressor genes when compared to normal tissue. | (31–34) |
| SPACA3🟀 | Sperm Acrosome membrane-associated protein 3/Cancer Testis Antigen 54 | Sperm surface membrane protein that may be involved in sperm-egg plasma membrane adhesion and fusion during fertilization. It could be a potential receptor for the egg oligosaccharide residue N-acetylglucosamine, which is present in the extracellular matrix over the egg plasma membrane. The processed form has no detectable bacteriolytic activity in vitro. | Unclear | SPACA3 expression shown to have a significant protective effect against distal recurrence in triple negative breast cancer (HR 0.471; p=0.006). SPACA3 expression was notably higher haematological malignancies with anti-SPACA3 IgG being detected in the sera of these patients. Moreover SPACA3 expression was notably higher in multiple myeloma cell lines which associated with negative prognostic factors and reduced overall survival. | (35–37) |
| IMPDH1 | Inosine Monophosphate Dehydrogenase 1/LCA11 | The protein encoded by this gene acts as a homotetramer to regulate cell growth. The encoded protein is an enzyme that catalyzes the synthesis of xanthine monophosphate (XMP) from inosine-5'-monophosphate (IMP). This is the rate-limiting step in the de novo synthesis of guanine nucleotides. | Oncogene | Notably higher expression of the IMPDH1 gene in breast cancer cells. Metabolomics data from small cell lung cancer cell lines identified abundant expression of IMPDH1 and 2 enzymes, inhibition of these enzymes reduced in vitro cell growth and improved survival in murine xenografts. Reduced overall survival was noted in patients with renal cell carcinoma (clear cell) whose tumours were high expressors of IMPDH1. | (38–40) |
| TXN2 | Thioredoxin 2/MT-TRX/COXPD29 | This nuclear gene encodes a mitochondrial member of the thioredoxin family, a group of small multifunctional redox-active proteins. The encoded protein may play important roles in the regulation of the mitochondrial membrane potential and in protection against oxidant-induced apoptosis and cell viability. | Tumour Suppressor Gene | TXN2 is known to inhibit transforming growth factor (TGF)-β-stimulated ROS generation independent of Smad signaling. TGF-β is a pro-oncogenic cytokine that induces epithelial–mesenchymal transition (EMT), which is a crucial event in metastatic progression. In particular, TXN2 inhibits TGF-β-mediated induction of HMGA2, a central EMT mediator, and fibronectin, an EMT marker. TXN2 expression has positively correlated however with higher stages of oral squamous cell carcinoma. | (41) |
| TFG | Trafficking from ER to Golgi Regulator/TRKT3 Oncogene/TRK-Fused Gene Protein | Plays a role in the normal dynamic function of the endoplasmic reticulum (ER) and its associated microtubules. Required for secretory cargo traffic from the endoplasmic reticulum to the Golgi apparatus. | Oncogene | TFG has been found to be rearranged in papillary thyroid and ROS-1 positive non-small cell lung cancers. In vitro studies have further determined TFG to be strongly linked to downstream Wnt pathway signalling. Enrichment of this gene was found in the proximity of Wnt co-receptor LRP6, and when silenced, significantly reduced Wnt/B-catenin signalling in cell culture. | (42,43) |
| PPP2R1A | Protein Phosphatase 2 Scaffold Subunit alpha/Serine Threonine Protein Phosphatase 2A | This gene encodes a constant regulatory subunit of protein phosphatase 2. Protein phosphatase 2 is one of the four major Ser/Thr phosphatases, and it is implicated in the negative control of cell growth and division. The PR65 subunit of protein phosphatase 2A serves as a scaffolding molecule to coordinate the assembly of the catalytic subunit and a variable regulatory B subunit. Upon interaction with GNA12 promotes dephosphorylation of microtubule associated protein TAU/MAPT. | Tumour Suppressor Gene | PPP2R1A expression has been observed at high frequency in endometrial and ovarian carcinomas; mutation of the protein itself enhances cancer cell migration through Src-Jnk-c-Jun signalling and over-expression of the wildtype protein also increases cell proliferation in vitro and tumour growth in vivo. PPP2R1A inactivation by recurrent missense mutations drives tumour growth and resistance to MEK inhibitors in solid tumours whereas allosteric activation of this enzyme results in anti-tumour activity mediated by dephosphorylation of MYBL2 resulting in cell cycle arrest. Mutations in this gene have be found in lung and oesophageal cancers. | (44–50) |
| 🟀Cancer Testis Antigen (CTAG) family |  |  |  |  |  |

**Supplemental References**

1. Miyamoto T, Sengoku K, Hasuike S, Takuma N, Hayashi H, Yamashita T, et al. Isolation and expression analysis of the human testis-specific gene, SPERGEN-1, a spermatogenic cell-specific gene-1. J Assist Reprod Genet. 2003 Feb;20(2):101–4.

2. Doiguchi M, Mori T, Toshimori K, Shibata Y, Iida H. Spergen-1 might be an adhesive molecule associated with mitochondria in the middle piece of spermatozoa. Dev Biol. 2002 Dec 1;252(1):127–37.

3. Ghafouri-Fard S, Ousati Ashtiani Z, Sabah Golian B, Hasheminasab S-M, Modarressi MH. Expression of two testis-specific genes, SPATA19 and LEMD1, in prostate cancer. Arch Med Res. 2010 Apr;41(3):195–200.

4. Wong KK, Hussain FA, Loo SK, López JI. Cancer/testis antigen SPATA19 is frequently expressed in benign prostatic hyperplasia and prostate cancer. APMIS Acta Pathol Microbiol Immunol Scand. 2017 Dec;125(12):1092–101.

5. Navarro-Romero MT, Muñoz M de L, Alcala-Castañeda E, Terreros-Espinosa E, Domínguez-de-la-Cruz E, García-Hernández N, et al. A novel method of male sex identification of human ancient skeletal remains. Chromosome Res Int J Mol Supramol Evol Asp Chromosome Biol. 2020 Dec;28(3–4):277–91.

6. López de la Oliva AR, Campos-Sandoval JA, Gómez-García MC, Cardona C, Martín-Rufián M, Sialana FJ, et al. Nuclear Translocation of Glutaminase GLS2 in Human Cancer Cells Associates with Proliferation Arrest and Differentiation. Sci Rep. 2020 Feb 10;10(1):2259.

7. Dias MM, Adamoski D, Dos Reis LM, Ascenção CFR, de Oliveira KRS, Mafra ACP, et al. GLS2 is protumorigenic in breast cancers. Oncogene. 2020 Jan;39(3):690–702.

8. Hu W, Zhang C, Wu R, Sun Y, Levine A, Feng Z. Glutaminase 2, a novel p53 target gene regulating energy metabolism and antioxidant function. Proc Natl Acad Sci U S A. 2010 Apr 20;107(16):7455–60.

9. Liu J, Zhang C, Lin M, Zhu W, Liang Y, Hong X, et al. Glutaminase 2 negatively regulates the PI3K/AKT signaling and shows tumor suppression activity in human hepatocellular carcinoma. Oncotarget. 2014 May 15;5(9):2635–47.

10. Szeliga M, Bogacińska-Karaś M, Różycka A, Hilgier W, Marquez J, Albrecht J. Silencing of GLS and overexpression of GLS2 genes cooperate in decreasing the proliferation and viability of glioblastoma cells. Tumour Biol J Int Soc Oncodevelopmental Biol Med. 2014 Mar;35(3):1855–62.

11. Szeliga M, Bogacińska-Karaś M, Kuźmicz K, Rola R, Albrecht J. Downregulation of GLS2 in glioblastoma cells is related to DNA hypermethylation but not to the p53 status. Mol Carcinog. 2016 Sep;55(9):1309–16.

12. Sijbrandi R, Fiedler U, Timmers HTM. RNA polymerase II complexes in the very early phase of transcription are not susceptible to TFIIS-induced exonucleolytic cleavage. Nucleic Acids Res. 2002 Jun 1;30(11):2290–8.

13. Scotto L, Narayan G, Nandula SV, Arias‐Pulido H, Subramaniyam S, Schneider A, et al. Identification of copy number gain and overexpressed genes on chromosome arm 20q by an integrative genomic approach in cervical cancer: Potential role in progression. Genes Chromosomes Cancer. 2008;47(9):755–65.

14. Hill SJ, Rolland T, Adelmant G, Xia X, Owen MS, Dricot A, et al. Systematic screening reveals a role for BRCA1 in the response to transcription-associated DNA damage. Genes Dev. 2014 Sep 1;28(17):1957–75.

15. Sha Y-W, Sha Y-K, Ji Z-Y, Mei L-B, Ding L, Zhang Q, et al. TSGA10 is a novel candidate gene associated with acephalic spermatozoa. Clin Genet. 2018 Apr;93(4):776–83.

16. Bao L, You B, Shi S, Shan Y, Zhang Q, Yue H, et al. Metastasis-associated miR-23a from nasopharyngeal carcinoma-derived exosomes mediates angiogenesis by repressing a novel target gene TSGA10. Oncogene. 2018 May;37(21):2873–89.

17. Jahani M, Shahlaei M, Norooznezhad F, Miraghaee SS, Hosseinzadeh L, Moasefi N, et al. TSGA10 Over Expression Decreases Metastasic and Metabolic Activity by Inhibiting HIF-1 in Breast Cancer Cells. Arch Med Res. 2020 Jan;51(1):41–53.

18. Wu D, Lin J, Zhu Y, Zhang H, Zhong Y. Expression of Testis-Specific Gene Antigen 10 (TSGA10) is Associated with Apoptosis and Cell Migration in Bladder Cancer Cells and Tumor Stage and Overall Survival in Patients with Bladder Cancer. Med Sci Monit Int Med J Exp Clin Res. 2019 Jul 16;25:5289–98.

19. Chen P, Wang X-L, Ma Z-S, Xu Z, Jia B, Ren J, et al. Knockdown of HMGN5 expression by RNA interference induces cell cycle arrest in human lung cancer cells. Asian Pac J Cancer Prev APJCP. 2012;13(7):3223–8.

20. Furusawa T, Rochman M, Taher L, Dimitriadis EK, Nagashima K, Anderson S, et al. Chromatin decompaction by the nucleosomal binding protein HMGN5 impairs nuclear sturdiness. Nat Commun. 2015 Jan 22;6:6138.

21. Gan Y, He L, Yao K, Tan J, Zeng Q, Dai Y, et al. Knockdown of HMGN5 increases the chemosensitivity of human urothelial bladder cancer cells to cisplatin by targeting PI3K/Akt signaling. Oncol Lett. 2017 Dec;14(6):6463–70.

22. Ma Q, Wang X, Wang H, Song W, Wang Q, Wang J. HMGN5 Silencing Suppresses Cell Biological Progression via AKT/MAPK Pathway in Human Glioblastoma Cells. BioMed Res Int. 2020;2020:8610271.

23. Yao K, He L, Gan Y, Liu J, Tang J, Long Z, et al. HMGN5 promotes IL-6-induced epithelial-mesenchymal transition of bladder cancer by interacting with Hsp27. Aging. 2020 Apr 21;12(8):7282–98.

24. Zhao J, Wang Y, Wu X. HMGN5 promotes proliferation and invasion via the activation of Wnt/β-catenin signaling pathway in pancreatic ductal adenocarcinoma. Oncol Lett. 2018 Sep;16(3):4013–9.

25. He J, Liu C, Wang B, Li N, Zuo G, Gao D. HMGN5 blockade by siRNA enhances apoptosis, suppresses invasion and increases chemosensitivity to temozolomide in meningiomas. Int J Oncol. 2015 Oct;47(4):1503–11.

26. Liu X, Ma W, Yan Y, Wu S. Silencing HMGN5 suppresses cell growth and promotes chemosensitivity in esophageal squamous cell carcinoma. J Biochem Mol Toxicol. 2017 Dec;31(12).

27. Wu J, Wang J. HMGN5 expression in bladder cancer tissue and its role on prognosis. Eur Rev Med Pharmacol Sci. 2018 Feb;22(4):970–5.

28. Viphakone N, Cumberbatch MG, Livingstone MJ, Heath PR, Dickman MJ, Catto JW, et al. Luzp4 defines a new mRNA export pathway in cancer cells. Nucleic Acids Res. 2015 Feb 27;43(4):2353–66.

29. de Anda-Jáuregui G, Velázquez-Caldelas TE, Espinal-Enríquez J, Hernández-Lemus E. Transcriptional Network Architecture of Breast Cancer Molecular Subtypes. Front Physiol [Internet]. 2016 Nov 22 [cited 2021 Jan 31];7. Available from: https://www.ncbi.nlm.nih.gov/pmc/articles/PMC5118907/

30. Türeci O, Sahin U, Koslowski M, Buss B, Bell C, Ballweber P, et al. A novel tumour associated leucine zipper protein targeting to sites of gene transcription and splicing. Oncogene. 2002 May 30;21(24):3879–88.

31. Stronach EA, Alfraidi A, Rama N, Datler C, Studd JB, Agarwal R, et al. HDAC4-regulated STAT1 activation mediates platinum resistance in ovarian cancer. Cancer Res. 2011 Jul 1;71(13):4412–22.

32. Jin K, Zhao W, Xie X, Pan Y, Wang K, Zhang H. MiR-520b restrains cell growth by targeting HDAC4 in lung cancer. Thorac Cancer. 2018 Oct;9(10):1249–54.

33. Wang Z, Qin G, Zhao TC. Histone Deacetylase 4 (HDAC4): Mechanism of Regulations and Biological Functions. Epigenomics. 2014 Feb;6(1):139–50.

34. LLeonart ME, Vidal F, Gallardo D, Diaz-Fuertes M, Rojo F, Cuatrecasas M, et al. New p53 related genes in human tumors: significant downregulation in colon and lung carcinomas. Oncol Rep. 2006 Sep;16(3):603–8.

35. Yousef S, Heise J, Lajmi N, Bartels K, Kröger N, Luetkens T, et al. Cancer-testis antigen SLLP1 represents a promising target for the immunotherapy of multiple myeloma. J Transl Med. 2015 Jun 20;13:197.

36. Mandal A, Klotz KL, Shetty J, Jayes FL, Wolkowicz MJ, Bolling LC, et al. SLLP1, a unique, intra-acrosomal, non-bacteriolytic, c lysozyme-like protein of human spermatozoa. Biol Reprod. 2003 May;68(5):1525–37.

37. Wang Z, Zhang Y, Mandal A, Zhang J, Giles FJ, Herr JC, et al. The spermatozoa protein, SLLP1, is a novel cancer-testis antigen in hematologic malignancies. Clin Cancer Res Off J Am Assoc Cancer Res. 2004 Oct 1;10(19):6544–50.

38. Huang F, Ni M, Chalishazar MD, Huffman KE, Kim J, Cai L, et al. Inosine Monophosphate Dehydrogenase Dependence in a Subset of Small Cell Lung Cancers. Cell Metab. 2018 Sep 4;28(3):369-382.e5.

39. Ouchida M, Kanzaki H, Ito S, Hanafusa H, Jitsumori Y, Tamaru S, et al. Novel direct targets of miR-19a identified in breast cancer cells by a quantitative proteomic approach. PloS One. 2012;7(8):e44095.

40. Ruan H, Song Z, Cao Q, Ni D, Xu T, Wang K, et al. IMPDH1/YB-1 Positive Feedback Loop Assembles Cytoophidia and Represents a Therapeutic Target in Metastatic Tumors. Mol Ther J Am Soc Gene Ther. 2020 May 6;28(5):1299–313.

41. Ishikawa F, Kaneko E, Sugimoto T, Ishijima T, Wakamatsu M, Yuasa A, et al. A mitochondrial thioredoxin-sensitive mechanism regulates TGF-β-mediated gene expression associated with epithelial-mesenchymal transition. Biochem Biophys Res Commun. 2014 Jan 17;443(3):821–7.

42. Roccato E, Miranda C, Ranzi V, Gishizki M, Pierotti MA, Greco A. Biological activity of the thyroid TRK-T3 oncogene requires signalling through Shc. Br J Cancer. 2002 Sep 9;87(6):645–53.

43. Colozza G, Jami-Alahmadi Y, Dsouza A, Tejeda-Muñoz N, Albrecht LV, Sosa EA, et al. Wnt-inducible Lrp6-APEX2 interacting proteins identify ESCRT machinery and Trk-fused gene as components of the Wnt signaling pathway. Sci Rep [Internet]. 2020 Dec 9 [cited 2021 Feb 1];10. Available from: https://www.ncbi.nlm.nih.gov/pmc/articles/PMC7726150/

44. Morita K, He S, Nowak RP, Wang J, Zimmerman MW, Fu C, et al. Allosteric Activators of Protein Phosphatase 2A Display Broad Antitumor Activity Mediated by Dephosphorylation of MYBL2. Cell. 2020 Apr 30;181(3):702-715.e20.

45. Jeong AL, Han S, Lee S, Su Park J, Lu Y, Yu S, et al. Patient derived mutation W257G of PPP2R1A enhances cancer cell migration through SRC-JNK-c-Jun pathway. Sci Rep. 2016 Jun 7;6(1):27391.

46. Zhu D, Kosik KS, Meigs TE, Yanamadala V, Denker BM. Galpha12 directly interacts with PP2A: evidence FOR Galpha12-stimulated PP2A phosphatase activity and dephosphorylation of microtubule-associated protein, tau. J Biol Chem. 2004 Dec 31;279(53):54983–6.

47. Hsieh F-S, Hung M-H, Wang C-Y, Chen Y-L, Hsiao Y-J, Tsai M-H, et al. Inhibition of protein phosphatase 5 suppresses non-small cell lung cancer through AMP-activated kinase activation. Lung Cancer Amst Neth. 2017 Oct;112:81–9.

48. O’Connor CM, Leonard D, Wiredja D, Avelar RA, Wang Z, Schlatzer D, et al. Inactivation of PP2A by a recurrent mutation drives resistance to MEK inhibitors. Oncogene. 2020 Jan;39(3):703–17.

49. Julien SG, Dubé N, Read M, Penney J, Paquet M, Han Y, et al. Protein tyrosine phosphatase 1B deficiency or inhibition delays ErbB2-induced mammary tumorigenesis and protects from lung metastasis. Nat Genet. 2007 Mar;39(3):338–46.

50. Zhao-feng W, Hong-bing LIU, Yan-wen Y a. O, Fang-fang C, Yong S. Expression of protein-tyrosine-phosphatase 1B in non-small-cell lung cancer and its prognostic significance. Natl Med J China. 2013 Oct 15;93(38):3027–30.
