# Supplementary material for: A highly predictive autoantibody-based biomarker panel for prognosis in early-stage NSCLC with potential therapeutic implications
Source: Br J Cancer. 2021 Nov 2;126(2):238–46. doi: 10.1038/s41416-021-01572-x (PMC8770460; doi:10.1038/s41416-021-01572-x)
Supplement: Supplementary file 9 — List of Supplementary Material [file 41416_2021_1572_MOESM9_ESM.docx]

**List of Supplementary Material**

Supplementary Table (S1).docx

Supplementary Methods (S2).docx

Supplementary Table (S3).docx

Supplementary Graph (S4).docx

Supplementary Table (S5).docx

Supplementary Table (S6).docx

Supplementary Figure (S7).docx

Supplementary Table (S8).docx
